# Expanding the Potential of Self-Assembled Silk Fibroin as Aerogel Particles for Tissue Regeneration

**DOI:** 10.3390/pharmaceutics15112605

**Published:** 2023-11-09

**Authors:** Beatriz G. Bernardes, Sara Baptista-Silva, Carlos Illanes-Bordomás, Rui Magalhães, Juliana Rosa Dias, Nuno M. F. Alves, Raquel Costa, Carlos A. García-González, Ana Leite Oliveira

**Affiliations:** 1 Universidade Católica Portuguesa, CBQF—Centro de Biotecnologia e Química Fina—Laboratório Associado, Escola Superior de Biotecnologia, Rua Diogo Botelho 1327, 4169-005 Porto, Portugal; bbernardes@ucp.pt (B.G.B.); sara.baptistadasilva@gmail.com (S.B.-S.); rsmagalhaes@ucp.pt (R.M.); 2 AerogelsLab, I+D Farma Group (GI-1645), Department of Pharmacology, Pharmacy and Pharmaceutical Technology, iMATUS and Health Research Institute of Santiago de Compostela (IDIS), Universidade de Santiago de Compostela, E-15782 Santiago de Compostela, Spain; carlosjavier.illanes@rai.usc.es; 3 Centre for Rapid and Sustainable Product Development, Instituto Politécnico de Leiria, 2430-028 Marinha Grande, Portugal; juliana.dias@ipleiria.pt (J.R.D.); nuno.alves@ipleiria.pt (N.M.F.A.); 4 Biochemistry Unit, Department of Biomedicine, Faculdade de Medicina, Universidade do Porto, 4200-319 Porto, Portugal

**Keywords:** aerogel, tissue engineering, particles and silk fibroin

## Abstract

A newly produced silk fibroin (SF) aerogel particulate system using a supercritical carbon dioxide (scCO_2_)-assisted drying technology is herein proposed for biomedical applications. Different concentrations of silk fibroin (3%, 5%, and 7% (*w*/*v*)) were explored to investigate the potential of this technology to produce size- and porosity-controlled particles. Laser diffraction, helium pycnometry, nitrogen adsorption–desorption analysis and Fourier Transform Infrared with Attenuated Total Reflectance (FTIR-ATR) spectroscopy were performed to characterize the physicochemical properties of the material. The enzymatic degradation profile of the SF aerogel particles was evaluated by immersion in protease XIV solution, and the biological properties by cell viability and cell proliferation assays. The obtained aerogel particles were mesoporous with high and concentration dependent specific surface area (203–326 m^2^/g). They displayed significant antioxidant activity and sustained degradation in the presence of protease XIV enzyme. The in vitro assessment using human dermal fibroblasts (HDF) confirm the particles’ biocompatibility, as well as the enhancement in cell viability and proliferation.

## 1. Introduction

Aerogels are promising nanostructured materials for biomedical applications, due to their high porosity (90–99.99%), large surface area, and ultra-lightness [1,2]. Aerogels are obtained from wet gel precursors through a proper drying technology (typically supercritical drying) able to remove the pore liquid with very low structural modifications [2,3]. Bio-based aerogels, i.e., from natural polymer sources like cellulose, collagen, alginate, or silk proteins, can provide advanced biomedical performances due to their native properties, such as density, porosity, biocompatibility, and biodegradability. These bioaerogels have been used in tissue engineering [2], biosensing [2], and therapeutic applications [4]. A desirable delivery system should ensure the stability of the incorporated bioactive agent, while enabling modified release profiles [5,6]. In this context, protein-based aerogels have high potential as drug carriers [7], as they present an adequate biological performance to promote the required physiological response, while providing a high loading capacity and tunable drug release [8]. 

Traditionally used as fiber in the textile industry, silk has found extensive application in the fields of biomedical engineering and pharmaceutical technology in the last decades [9]. It exhibits an outstanding strength-to-density ratio, thanks to the plentiful highly organized β-sheet crystallites it contains, complemented by the supporting amorphous bridges that contribute to its lateral strength and elasticity [10]. Silk fibroin (SF), the main structural protein of silk, is mainly composed of hydrophobic β-sheet crystallites and hydrophilic amorphous domains. The crystalline domains are composed of heavy- and light-chain polypeptides that give SF a rare combination of properties, such as strength, modulus, toughness, light weight and elasticity, oxygen and water vapor permeability, tailorable degradability, signaling molecules stabilization ability and biocompatibility [10,11,12]. 

Porous SF materials have been developed in different formats, such as scaffolds [9,13,14], nanofibers [15], microcarriers [16], hydrogels [17], and composites [18,19,20,21] which can be applied for wound healing treatment with tissue regenerative properties. Mallepally et al. [13] produced SF aerogel scaffolds for tissue engineering applications with high surface area using a scCO_2_-assisted drying technique. Marin et al. [22] produced SF aerogels as drug delivery systems for the extended release of ibuprofen using scCO_2_. Dodel et al. [15] prepared SF nanofibers as knitted and angular samples using the gap electrospinning method, followed by freeze-drying method, for regenerative medicine application. A biocompatible, biodegradable, and antibacterial SF/chitin 3D nanocomposite scaffold incorporating silver and TiO2 nanoparticles was fabricated via the freeze-drying method for successful wound dressing application by Mehrabani et al. [18,19]. SF aerogels have been developed for other applications like bone tissue regeneration [23] and textiles for personal thermal management [24], among others. Goimil et al. [23] prepared a scaffold containing PCL, SF aerogel microparticles, and dexamethasone for bone tissue regeneration. An emulsion-gelation of paraffin oil and ultrasound was used to produce the SF microparticles, and different gelation promoters (ethanol and CO_2_ sparging) were tested to obtain the SF gel microparticles, followed by solvent exchange and scCO_2_ [23]. 

The present research work finds its motivation on the recognized need for innovative material systems that can significantly contribute to stimulate tissue regeneration in applications such as in the context of wound healing. The healing process of an injury comprises a series of steps: hemostasis, inflammation, proliferation/maturation, and remodeling. During wound healing, a fluid (exudate) can be produced as a natural response towards healing. However, its excessive production can be detrimental, as it can promote bacterial growth, delaying the inflammatory/swelling phase, which represents a challenge for wound management [2]. Various cell types play crucial roles in the wound healing process according to its phase. The most common cells to be recruited during wound healing are fibroblasts, endothelial, and macrophages during hemostasis; monocytes, neutrophils, and macrophages during the inflammation phase; and keratinocytes, fibroblasts, and endothelial cells during the proliferation phase [2,25]. Keratinocytes, which are the primary constituents of the epidermis, rapidly respond when a skin injury occurs. Furthermore, they initiate the activation of fibroblasts by secreting interleukin 1 [26]. Fibroblasts serve as the primary connective tissue cells in the body and are responsible for collagen production in the extracellular matrix (ECM), preservation of physiological functions, and wound repair [25,27]. Moreover, some studies revealed their remarkable heterogeneity and adaptability, delineating discernible subtypes characterized by distinctive phenotypic and functional attributes [27]. This phenotypic variance in fibroblasts is contributory to the distinct anatomical sites and diverse biological processes [27]. Endothelial cells, on the other hand, play a pivotal role in the formation of new blood vessels [2]. Chronic wound healing is often hindered by the compromised migration of keratinocytes to the wound site, indicating a failure to successfully complete either one of the two potential pathways: activation or differentiation [28].

Due to the complexity of the wound healing process, conventional methods of wound healing often encounter the above-mentioned obstacles and, consequently, there is a growing demand for advanced biomaterials capable to manage the swelling effectively, enhancing the healing process and facilitating tissue regeneration. A newly produced silk fibroin aerogel particulate system, using a scCO_2_-assisted drying technology is herein proposed to be applied in biomedical applications. These aerogels are expected to exhibit desirable physicochemical properties and to promote wound healing, tissue repair, and other therapeutic responses due to their preserved textural properties, and ability to support cell viability, adhesion, and proliferation [3]. For SF particle production, absolute ethanol was used to stabilize and further dry SF gel particles. Laser diffraction, helium pycnometry, nitrogen adsorption−desorption analysis (Brunauer−Emmet−Teller (BET) and the Barrett−Joyner−Halenda (BJH) methods) and Fourier Transform Infrared with Attenuated Total Reflectance (FTIR-ATR) spectroscopy were performed to characterize the material physicochemical properties. The enzymatic degradation profile of the SF aerogel particles was evaluated by soaking them in protease XIV solution. The in vitro cell viability, adhesion, and proliferation were also assessed using human dermal fibroblasts (HDF), a cell type which plays a crucial role in biomedical applications, particularly in wound healing, where they are key contributors to the formation of new tissue and extracellular matrix, promoting the regeneration and repair of damaged skin.

## 2. Materials and Methods

### 2.1. Materials and Reagents

SF was extracted from *Bombyx mori* (*B. mori*) cocoons provided by the Portuguese Association of Parents and Friends of Mentally Disabled Citizens (APPA-CDM, Portugal). Sodium carbonate anhydrous (Na_2_CO_3_) (≥99.8%), lithium bromide (LiBr) (≥99%), and absolute ethanol (≥99.8%) were obtained from Honeywell (Wabash, IN, USA). Span^®^ 80 (428.62 g/mol) was purchased from Sigma Aldrich (St. Louis, MO, USA). 

### 2.2. Preparation of Silk Fibroin Aerogel Particles

#### 2.2.1. Silk Fibroin Extraction

SF was extracted from silkworm *B. mori* cocoons according to a previous procedure [29]. Initially, the cocoons were boiled in a solution of 0.02 M of Na_2_CO_3_ for 20 min and washed three times with boiled water for 20 min each, followed by an SF mesh rinse and SF drying in the oven at 40 °C overnight. To produce an aqueous SF solution (pure solution), degummed fibers were dissolved in a 9.3 M LiBr solution at 60 °C for 4 h, with a ratio of SF:LiBr of 1:5 (wt./v). The dissolved SF was dialyzed against distilled water, using a benzoylated dialysis tubing (molecular weight cut-off: 2000 Da; Sigma Aldrich, St. Louis, MO, USA), for 72 h to remove the salt and with water changes two times a day.

#### 2.2.2. Silk Fibroin Gel Particles Production

**Figure 1:**
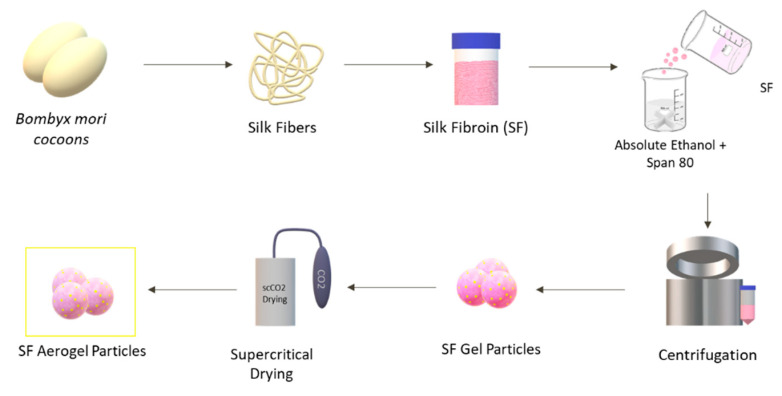
Schematic illustration of the procedure used for preparing SF aerogel particles.

To produce the particles (Figure 1), varying concentrations (3%, 5%, and 7% (*w*/*v*)) of SF aqueous solutions were carefully added drop by drop to a solution of absolute ethanol and the surfactant Sorbitan oleate (Span^®^ 80). The mixture, prepared with a 2:1 (*v*/*v*) ratio of absolute ethanol, also contained the surfactant Span^®^ 80 at a concentration of 3 wt.% relative to the weight of the SF solution. SF aqueous solutions were precipitated to the mixture with a stirring velocity of 600 rpm. SF droplets formed gel particles when in contact with the ethanolic solution [30].

#### 2.2.3. Solvent Exchange

The SF gel particles were submitted to a centrifugation process for water removal for 15 min at 5000 rpm. The solvent was exchanged to absolute ethanol (40 mL) immediately. After at least 3 h, a second solvent exchange with a similar volume of absolute ethanol was carried out to eliminate any remnant of water in the gel particles, obtaining alcogel particles. This process is necessary because CO_2_ and water at supercritical conditions of CO_2_ (>31.1 °C; >73.8 bar) exhibit very low affinity [1,31]. For that reason, it is extremely necessary to remove all the water present in the SF hydrogel particles, avoiding surface tension forces and capillary pressures that can cause a structural collapse of the gel network [32]. 

#### 2.2.4. Silk Fibroin Aerogel Particles Production

To obtain the SF aerogel particles (SF-Ap), alcogel particles were submitted to a scCO_2_-assisted drying process (120 bar, 39 °C, 3.5 h) for the extraction of ethanol, following a previous procedure [1]. 

### 2.3. Physicochemical Properties 

The size of SF particles (alcogel and aerogel) was determined with a particle sizer analyzer (Mastersizer 3000E, Malvern, UK) by the Mie theory method. Measurements were run at room temperature for six replicates. The refraction index of ethanol and SF dispersion were 1.36 and 1.56, respectively, with an absorption index of SF of 0.1. A dispersant consisting of 96 vol.% ethanol solution was used. The particle size was determined by measuring the diameter of particles introducing a volume of SF gel particles into the dispersant and dispersing the particles with a stirring velocity of 3500 rpm. Particle size distributions were reported by parameters based on the maximum particle size for a given percentile of the sample volume (Dv10, Dv50, and Dv90). The Dv50 is the maximum particle diameter below which 50% of the sample volume exists, also known as the average particle size by volume. Similarly, the Dv10 and Dv90 values indicate that 10% and 90%, respectively, are contained in droplets smaller than these values [33]. The spread of the droplet size distribution was determined by calculating the span with Equation (1):

(1) $$Span=\frac{Dv90-Dv10}{Dv50}$$

The surface morphology of SF-Ap was analyzed by scanning electron microscopy (SEM). SF-Ap were placed on observation pins, covered with double-sided adhesive carbon tape (NEM tape; Nisshin, Japan), sputter-coated with a thin layer (9–12 nm) of gold/palladium (Polaron, Bad Schwalbach, Germany), and observed using a Phenom XL G2 SEM (Thermo Fischer Scientific—FEI, Eindhoven, the Netherlands). The micrographs were obtained at an accelerating voltage of 10 kV at different magnifications, utilizing the secondary electron detector (SED).

The SF-Ap diameter was also determined using a CKC53 optical microscope equipped with an EP50 camera and EPview image analysis software v.1.3 (Olympus, Tokyo, Japan). In the particle size analysis conducted for this study, microscopy techniques were employed to measure the diameters of 50 individual particles for each concentration. The particles were sorted in ascending order based on their diameters. Subsequently, the cumulative percentage of particles was calculated for each diameter value. To determine the characteristic diameters Dv10, Dv50, and Dv90, the particle diameters corresponding to the 10th, 50th, and 90th percentiles of the cumulative percentage were identified. SF-Ap’s skeletal density ($\rho_{skel}$) was determined using helium pycnometry (MPY-2, Quantachrome, Delray Beach, FL, USA) at 25 °C and 1.03 bar from three replicates. The tapped density ($\rho_{tapped}$) of SF-Ap was calculated as the ratio between the weight of a certain amount of SF aerogel particles and the tapped volume. A tapped density tester equipment was used with 22–23 strokes per minute to determine the tapped volume. In 3D structures, various types of unit cell packing arrangements exist and the packing efficiency of each arrangement is dependent on the size and shape of the constituent atoms or molecules. Aerogels, which are highly porous structures, are formed through the arrangement of their constituent particles or molecules in a closely packed pattern. Moreover, the packing efficiencies of the aerogel particles after the tapping process were estimated as analogous to cubic close packing, at around 74%, resulting in a relatively low percentage of empty space within the unit cell. Therefore, aerogels exhibit a high degree of porosity within the particles along with roughly 26% of the total volume of the particle bed being occupied by empty space (i.e., interparticle voids) [34]. This information was taken into consideration to determine the envelope density ($\rho_{env}$) of SF-Ap, and 26% of these empty spaces were removed from the total volume occupied by tapped SF-Ap.

The overall porosity (ε) of the dried gels was expressed in percentage and calculated according to Equation (2):

(2) $$\varepsilon =\left(1-\frac{\rho_{env}}{\rho_{skel}}\right)\times 100\;$$

The textural and morphological properties of SF-Ap were characterized by nitrogen adsorption–desorption analysis (ASAP 2000, Micromeritics, Norcross, GA, USA) [1]. The Brunauer–Emmet–Teller (BET) and the Barrett–Joyner–Halenda (BJH) methods were applied to calculate the specific surface area (aBET) and the pore size distribution, respectively. The overall specific pore volume (Vp,BJH) and the mean pore diameter (Dp,BJH) were also obtained from the BJH method. The specific mesopore volume (V_mes_) was obtained from the cumulative BJH-pore volume profiles of the aerogels in the mesopore range (2–50 nm). The specific volume occupied by the macropores (V_mac_) in the aerogels was calculated as the difference between the total specific pore volumes of the aerogels (i.e., the inverse of the envelope density) and the specific pore volume occupied by mesopores (V_mes_) [1].

FTIR-ATR spectroscopy was used to investigate the secondary structure formation and conformation of SF-Ap and to analyze the chemical structure and crystallinity of the produced particles. FTIR-ATR analysis of SF-Ap was performed in a Perkin Elmer spectrometer, spectrum 100 (Waltham, MA, USA) that was equipped with an attenuated total reflectance (ATR) sampling accessory (PIKE Technologies, Beaconsfield, UK) and a diamond/ZnSe crystal. For each sample, a measurement of 32 scans was collected at a resolution of 4 cm^−1^, which was acquired over a wavenumber range of 600–4000 cm^−1^. In addition, baseline point adjustment and spectra normalization were performed.

### 2.4. Antioxidant Activity

The antioxidant activity of SF-Ap was assessed by 2,2-azinobis-3-ethylbenzothiazoline-6-sulfonic acid (ABTS) radical as a photometric method and oxygen radical absorbance capacity (ORAC) as a fluorometric assay. ABTS is based on the reduction by the presence of antioxidant compounds of a well-known metastable radical (ABTS•+), instead, ORAC measures the scavenging of the peroxyl radical AAPH by antioxidant compounds. The antioxidant potential of SF-Ap was for the first time evaluated under physiological protease XIV (Streptomyces griseus, 3.5 U/mg) degradation during 72 h at 37 °C. Aliquots at different time points were taken (24 h, 48 h, and 72 h). Both methods were performed in a 96-well microplate, following the procedure described by Cunha et al. 2020 [35]. ABTS radical cation (ABTS•+) was produced from the reaction of 7 mM 2,2-azinobis (3-ethylbenzothiazoline-6-sulfonic acid) diammonium salt and 2.45 mM potassium persulfate (both from Sigma-Aldrich, St. Louis, MO, USA). The reaction was obtained using 180 µL of ABTS•+ working solution with 20 µL of sample or Trolox as standard calibration curve (25–175 μM), at an absorbance of 0.70 ± 0.02 at 734 nm. The absorbance profile was measured in a multidetection plate reader (Synergy H1, Winooski, VT, USA) controlled by the Gen5 Biotek software version 3.04. The scavenging activity was expressed as a % reduction in absorbance for the control. Regression equations between SF-Ap ABTS scavenging properties and Trolox concentration were calculated, and the results were expressed as μmol TE (Trolox equivalent)/mL. The ORAC reaction was performed in a multidetection plate reader (Synergy H1; BioTek Instruments, Winooski VT, USA) with excitation and emission wavelengths of 485 nm and 528 nm, respectively. Trolox (1–8 µM, final concentration in well) was also used as the standard for the calibration curve. The results were expressed in µmol TE (Trolox equivalent)/mL for SF-Ap samples.

The stability of the SF-Ap was determined by enzymatic degradation tests. Protease XIV (Streptomyces griseus, 3.5 U/mg) was dissolved in PBS pH 7.4 solution at 1 U/mL. Samples were also incubated in PBS solution to be used as controls. The initial wet weight of each sample was measured after hydration in PBS solution for 3 h at 37 °C, and then each scaffold was immersed in 5 mL of protease solution or fresh PBS solution. The study was conducted at 37 °C for a time ranging from 6 h to 28 days. Cell strainers with 1 µm porosity were used as support for SF-Ap. At the end of each time point, the PBS or PBS with protease was removed using a cell strainer connecting ring. Before weighing, samples were gently blotted with filter paper to remove the excess liquid. The degradation ratio at the time point was calculated using the following Equation (3):

(3) $$weightloss\left(\%\right)=\frac{w_{i}-w_{t}}{w_{i}}\times 100$$

where $w_{i}$ is the initial wet weight of the hydrogel and $w_{t}$ is the wet weight tested at each point.

### 2.5. In Vitro Cell Viability and Cell Behavior

#### 2.5.1. Cell Seeding and Particles Incorporation

The biocompatibility of the developed microparticulate aerogel was evaluated by direct contact with human skin cells, namely fibroblasts, following ISO10993-5:2009 guidelines [36]. Human dermal fibroblasts (HDF) (Innoprot, Bizkaia, Spain) were seeded in 96-well plates to assess cell viability at a density of 1 × 10^5^ cells per cm^2^. Cell adhesion and morphology of SF-Ap were observed by SEM and confocal microscopies after 1, 3, and 7 days using non-adherent 24-well plates with tissue culture polystyrene coverslips (TCPS) (*n* = 3). The cell culture medium used was Dulbecco’s Modified Eagle’s Medium (DMEM-Dulbecco’s Modified Eagle Medium; Gibco™ DMEM, high glucose), supplemented with 10% fetal bovine serum (FBS; BioWest), and 1% of penicillin−streptomycin solution (Lonza, Basel, Switzerland). The plates were then incubated at 37 °C in a humidified atmosphere containing 5% CO_2_ for 24 h. SF particles at concentrations of 3%, 5%, and 7% (*w*/*v*) were sterilized using UV radiation for 20 min. Subsequently, they were suspended in a complete cell culture medium (5 mg/mL) and added to each well in triplicate. 

#### 2.5.2. MTT and BrdU Incorporation Assays

MTT (methyl thiazolyl tetrazolium) assay and in situ detection kit BrdU (Bromodeoxyuridine) quantification were used to evaluate the cell viability and proliferation of the cells. Prior to incorporating the particles into the wells, the cells in the BrdU plates were incubated with a BrdU labelling solution. Samples were collected after 1, 3, and 7 days for MTT assay to assess cell viability and in situ detection kit BrdU quantification to measure cell proliferation. The study included negative and positive controls, as well as blanks of DMEM medium and DMEM with aerogel particles.

The MTT test measures cell viability by converting a tetrazolium salt to a formazan compound in living cells, which is proportional to the number of viable cells. The resulting precipitate is solubilized in DMSO, and the absorbance is read at the wavelength of 570 nm using a microplate reader (Synergy Mx, Biotek, Santa Clara, CA, USA). 

BrdU is often used as a marker for cell proliferation studies. When cells are exposed to BrdU, the molecule is incorporated into newly synthesized DNA during the cell cycle. 

#### 2.5.3. Cell Morphology

For SEM analysis, the TCPS constructs were washed with PBS and fixed with a 2.5% glutaraldehyde solution in PBS pH 7.4 for 30 min. The samples were then washed twice with water, and dehydrated by immersing them (for a minimum of 10 min) in increasing concentrations of ethanol solutions (10%, 30%, 50%, 70%, 90%, and 100% (*v*/*v*)). Afterwards, samples were dried by dripping hexamethyldisilazane on top and immediately evaporating under a gentle stream of nitrogen. SF-Ap were placed on observation pins, covered with double-sided adhesive carbon tape (NEM tape; Nisshin, Japan), sputter-coated with a thin layer (9−12 nm) of gold/palladium (Polaron, Germany), and observed using a Phenom XL G2; Thermo Fischer Scientific (Eindhoven, Netherlands) SEM. The micrographs were obtained at an accelerating voltage of 5 kV at different magnifications, utilizing the secondary electron detector (SED).

Confocal analyses were performed to evaluate the cell morphology and fibronectin deposition for the studied time-points (1, 3, and 7 days of cell culture). The samples were stained for filamentous actin (F-actin), nuclei (DAPI), and fibronectin (FN). Briefly, samples were washed with PBS and fixed for 30 min in a 4% formalin solution at room temperature (RT). After fixation, the samples were washed with water and stored in sterile ultrapure water. Prior to observation, samples were permeabilized with 0.2% Triton X-100 (Sigma) for 7 min and then incubated for 1 h with 1 wt.% bovine serum albumin (BSA, Merck City, NJ, USA) in PBS. For FN staining, samples were incubated overnight at 4 ºC with rabbit anti-fibronectin (f3648, Sigma-Aldrich, Saint Louis, MO, USA, 1:300) and then with the goat anti-rabbit secondary antibody Alexa Fluor^®^ 488 F(ab’)2 fragment (Molecular Probes-Invitrogen, Eugene, OR, USA, 1:2000, 2 h at RT). After this, samples were incubated with the conjugated probe phalloidin/Alexa Fluor^®^ 594 (Molecular Probes-Invitrogen, Eugene, OR, USA, 1:40, 1 h at RT) for F-actin staining. Samples were subsequently washed three times with the PBS solution and nuclei were counterstained with 40,6-diamidino-2-phenylindole dihydrochloride (DAPI, Sigma-Aldrich, Saint Louis, MO, USA, 0.1 mg/mL) in vectashield (Vector laboratories, Newark, CA, USA), just before visualization. The stained samples were further observed under laser scanning confocal microscopy–Zeiss LSM900 confocal microscope (Zeiss, Jena, Germany). 

The controls used in the experiment included DMEM and human dermal fibroblasts (HDFs) cultured in DMEM as blank and negative controls, respectively.

### 2.6. Statistical Analysis

Quantitative data were subjected to an analysis of variance (one-way ANOVA) followed by a post hoc Tukey’s test, using a level of significance (*p*) of 0.05. Statistical analyses were performed using GraphPad Prism v. 9.3.1 (GraphPad Software, La Jolla, CA, USA).

## 3. Results and Discussion 

### 3.1. Morphological and Physicochemical Properties of SF Particles

**Figure 2:**
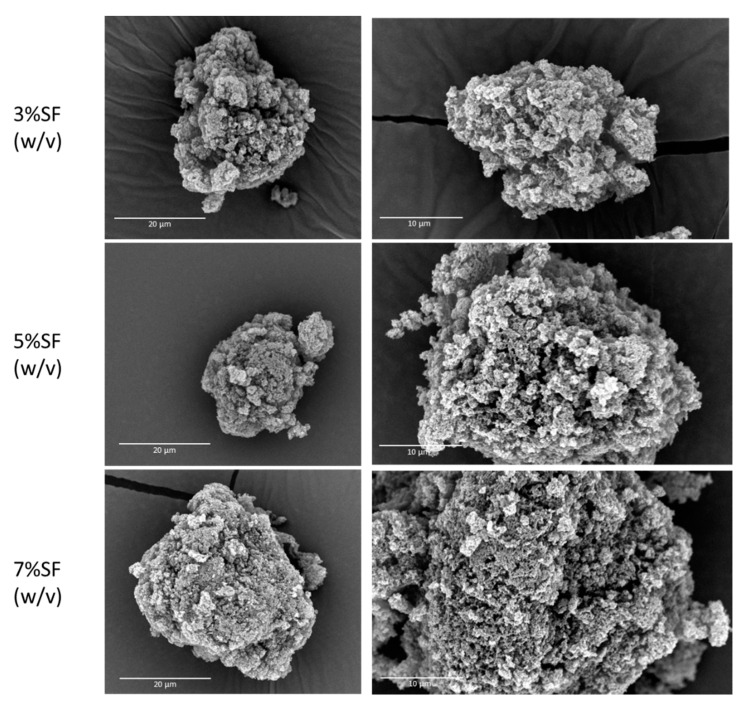
SEM micrographs of SF-Ap with 3%, 5%, and 7% SF (*w*/*v*).

The characterization of the SF particles and the influence of SF concentration in particle size distribution was studied by laser diffraction (Table 1). The SF concentration in alcogel particles had a direct impact on the size and distribution of the gel particles. Specifically, an increase in SF concentration led to an increase in particle diameter and dispersion (span in Table 1), which was attributed to the increased viscosity of the solution with SF concentration. The same trend was not observed in aerogel particles as there was an increase of Dv50 of 3% and 7% SF-Ap. These results with aerogels were attributed to the agglomeration of particles. To confirm this measurement artefact, SF-Aps were measured by analysis of images obtained with an optical microscope as an alternative method. In this case, the diameter of the aerogel particles measured by laser diffraction and microscopy (Table 1) showed some discrepancies, possibly due to the presence of particle agglomerates in the laser diffraction method. Accordingly, particle diameters of aerogels obtained by microscopy analysis were used to accurately determine the shrinkage volume of aerogels. After the supercritical drying, the particles preserved their structure, with an overall volume shrinkage of 8.0% ± 4.0% concerning the Dv50 of alcogel particles and average diameter of SF-Ap. The morphology of the SF aerogel particles (Figure 2) confirms the increase in particle size related to a higher concentration of the precursor SF solution.

The density and porosity of SF-Ap (Table 2) were similar among the tested concentrations. Additionally, the SF concentration did not influence the textural properties determined by nitrogen adsorption–desorption analysis (Table 3). All SF-Ap had high specific surface areas (203–326 m^2^/g) and pore volumes (1.53–2.53 cm^3^/g). These SF aerogel particles have textural properties very similar to those exhibited in the literature [13,22]. The concentration of SF affected the textural properties of the aerogels, as Mallepally et al. proposed [13]. They argued that higher SF concentrations would result in higher surface areas due to a more porous protein network. However, our results did not agree with this hypothesis, as the 7% SF aerogels had lower porosity and surface area than the other concentrations. According to the International Union of Pure and Applied Chemistry (IUPAC) adsorption–desorption isotherms classification, aerogels are classified as type IV, considering aerogels as primarily mesoporous materials with interconnected cavities that cause the hysteresis loop and a high volume of nitrogen adsorbed [1,37]. The pore size of SF-Ap (24–27 nm) is accordingly classified in the mesoporous range. The pore diameter obtained in this study was found to be higher than those reported for silk aerogel microparticles and monoliths [13,23]. This difference can be attributed to the different particle production methods (emulsion-gelation [23] and droplets in gaseous phase (vibrating nozzle, mechanical cutting, spraying) [38]) and gelation promoter (CO2-assisted gelation [13,39], crosslinking induced gelation (ethanol) [23], and salt-leaching method [13]) used with respect to the literature. The contribution of the mesopore and macropore volumes to the total volume was also studied (V_mes_ and V_mac_ in Table 3, respectively). Results indicate that the macropore volume is highly predominant (above 87% in all cases), suggesting that these structures could promote cell migration and drug diffusion when in contact with body fluid [40,41]. Examining the SEM images (Figure 2), SF-Ap does not exhibit any clearly visibly closed pores. Additionally, the results from nitrogen adsorption–desorption analysis demonstrate high specific surface areas and porosities, suggesting interconnectivity in this case.

**Figure 3:**
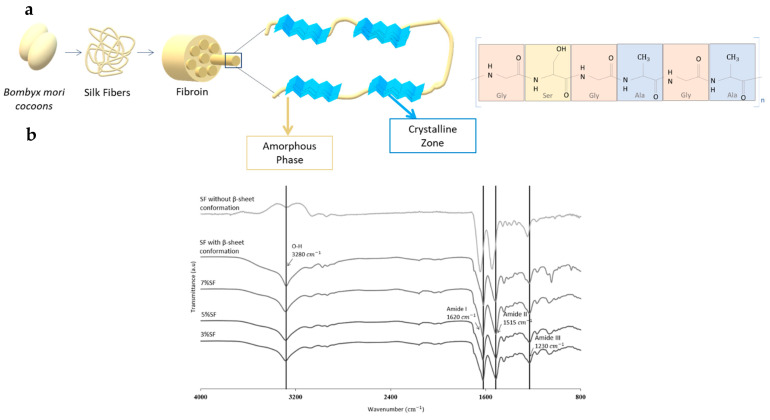
(**a**) Illustrative depiction of silk fibroin structure: Encompassing the silkworm thread, fibril’s overall organization, silk fibroin polypeptide chains, and amino acid composition. (**b**) FTIR-ATR spectra of SF-Ap with 3%, 5%, and 7% SF (*w*/*v*) and SF controls: SF without and with β-sheet conformation.

FTIR-ATR analysis of SF-Ap (Figure 3) provided valuable insights into the structural changes occurring in SF gels in the presence of ethanol. 

SF comprises a heavy and light chain connected by a single disulfide bridge as well as a glycoprotein named P25 (Figure 3a) [10,42,43]. SF is primarily composed of glycine (Gly) at 43%, alanine (Ala) at 30%, and serine (Ser) at 12%. Within the hydrophobic domains of the heavy chain, there is a repetitive hexapeptide sequence that has the ability to form stable anti-parallel β-sheet crystalline structures [42]. In contrast, the amino acid sequence of the light chain (L-chain) is non-repetitive, rendering it more hydrophilic and relatively elastic [42]. SF primarily exhibits two main crystal structures, silk I and silk II. Silk I adopts a non-random coil structure, and efforts to spin silk fibers directly from random coil-soluble fibroin have yielded weak results. The optimal formation of silk II involves the conversion of silk I into a nucleus for β-sheet formation [43]. 

The spectra obtained in Figure 3b confirm the presence of the main characteristic bands of SF assigned to β-sheet structure, in the amide I region 1600–1690 cm^−1^ (C=O stretching) and amide II region 1480–1575 cm^−1^ (C-N stretching) [14]. The untreated SF exhibited mostly an amorphous structure (1541 cm^−1^) with silk I structure (1646 cm^−1^). In the treated SF and the SF-Ap spectra it is possible to observe a peak shift to 1620 cm^−1^, indicating an increase of silk II β-sheet content with a simultaneous reduction of silk I content with bands in the range 1610–1630 cm^−1^ [14]. The increase in β-sheet content (silk II) is accountable for the hydrophobic nature of regenerated silk [44], and consequently of SF-Ap. In addition, the positions of these bands indicate the presence of the β-sheet (1620 cm^−1^) and random coil (1515 cm^−1^) conformations of the protein material for amide I and amide II, respectively [45]. The presence of the β-sheet conformation in the aerogel particles confirmed that in the presence of ethanol SF adopts a more stable conformation, leading to gel formation [46]. This gelation process with β-sheet structure formation can be beneficial for enhancing the mechanical (i.e., strength and stability) and biological properties of the aerogel particles [47].

### 3.2. Degradation Behavior and Antioxidant Activity

**Figure 4:**
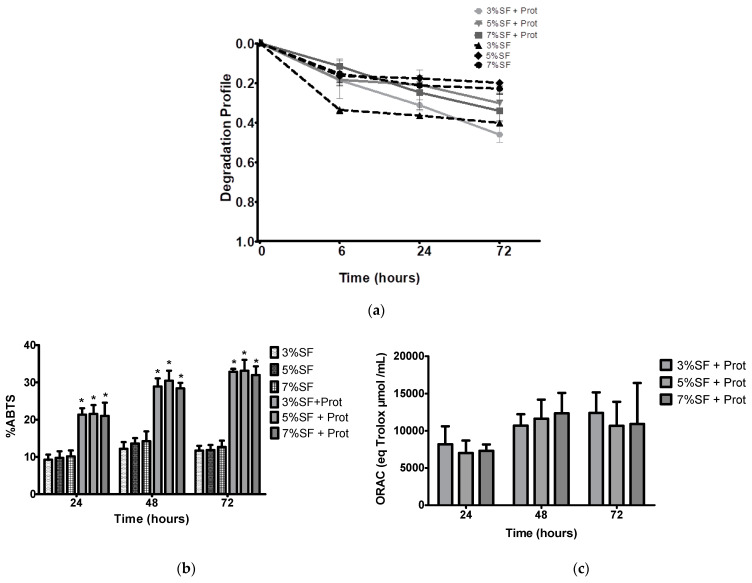
(**a**) Degradation of SF-Ap with PBS and with protease XVI during 1680 h, all of which occurred at 37 °C (*p* < 0.05). (**b**) SF-Ap antioxidant activity by ABTS (*p* < 0.05) and (**c**) by ORAC method obtained after physiological protease degradation during 24, 48, and 72 h at 37 °C. * The differences from the SF-Ap with protease with SF-Ap with PBS comparing of each time-point were significant at *p* < 0.05.

The in vitro degradation of materials is an important factor for biomedical applications. The antioxidant activity of a material is also relevant to prevent oxidative stresses and free radicals that can cause damage to cells and tissues, and this can be modulated during degradation. In vitro degradation tests can simulate the conditions that the material will encounter in the body and determine its biocompatibility, safety, and bioactive behavior. Based on the results depicted in Figure 4a, significant differences are observed at the 6 h time point between the 3% SF particles incubated with protease and those incubated with PBS. SF has been reported to exhibit significant antioxidant activity due to the presence of specific amino acids which possess strong radical scavenging properties [48]. The abundance of other chemical groups (hydroxyl and carboxyl) also contributes to the use of SF as a highly promising natural antioxidant [48]. In this study, we assess the antioxidant capacity of SF-Ap using two different methodologies (i.e., ABTS and ORAC) simultaneously under physiological protease XIV (3.5 U/mg) degradation after 72 h at 37 °C. This study follows the previous rationale of the group to a different silk protein ca. sericin, in which the antioxidant potential was evaluated concerning the degradation products, to evaluate the sustained release and bioactivity over time [17]. Our results concerning the enzymatic degradation showed an increase of SF-Ap antioxidant activity (Figure 4b,c). After 72 h the maximum activity was obtained, which may be related to the release of the phenolic and amino groups that after being released were still functional and may keep the radical scavenging ability [17]. The degradation rate of all tested groups was superior in the presence of protease XIV in comparison to the control condition (PBS solution) since SF is prone to proteolytic degradation. There are significant differences in the percentages of SF-Ap incubated with protease compared to those incubated with PBS at each time point in the ABTS assay (Figure 4b). The control groups did not exhibit any detectable antioxidant activity as assessed by ORAC. Based on the results obtained, the degradation rate of SF was not significantly affected by the concentration of SF in the presence of protease. This could be attributed to the higher release profile in this range, and to the higher surface area plus specific pore volume of these SF particles concentrations. Other different studies in which the effect of SF was tested on membranes after physiological degradation, showed the same evidence for the lowest protein concentrations in which 5% turmeric extract exhibited the highest antioxidant capacity in release media [49]. In another report, the antioxidant profile of SF was tested in hydrogels and evaluated by a similar radical inhibition assay method using the 2,2-diphenyl-1-picrylhydrazyl (DPPH), over the same period ca. 72 h. The DPPH radicals were instantly inhibited also in a concentration and time-dependent manner. The antioxidant activity of SF in this study was saturated after 24 h [50]. This sustained effect that we also found and that was higher in our study, is a desired property to be used in dynamically regulated living systems. Different data report the antioxidant activity of collagen-coated silk fibroin nanofibers to be used in wound healing and tissue regeneration. In this work the antioxidant activity of nanofibers was improved by the addition of two small molecules: sinomenine hydrochloride (SH) and kaempferol hydrate (KH) [51]. The combined system, due to its great antioxidant performance, was shown to minimize inflammation and enhanced collagen deposition. These data may impact the oxidation and help to control the inflammatory processes, as key factors for healing and tissue regeneration events. The biological cascade produces too many free radicals, which can harm proteins, lipids, and ECM components. The promising antioxidant properties of SF-Ap may contribute to accelerate and improve the healing process [17].

### 3.3. In Vitro Biocompatibility

The biocompatibility evaluation of SF-Ap was conducted using HDF cells. Fibroblasts are cells found in the dermis and are responsible for producing extracellular matrix components, such as collagen, that provide strength and stability to the healing tissue [52]. In addition, fibroblasts also play a role in remodeling the extracellular matrix and regulating the immune response during wound healing [2,52]. Understanding the interaction between HDF with SF-Ap is critical to understand how these particles promote wound healing and tissue regeneration. 

**Figure 5:**
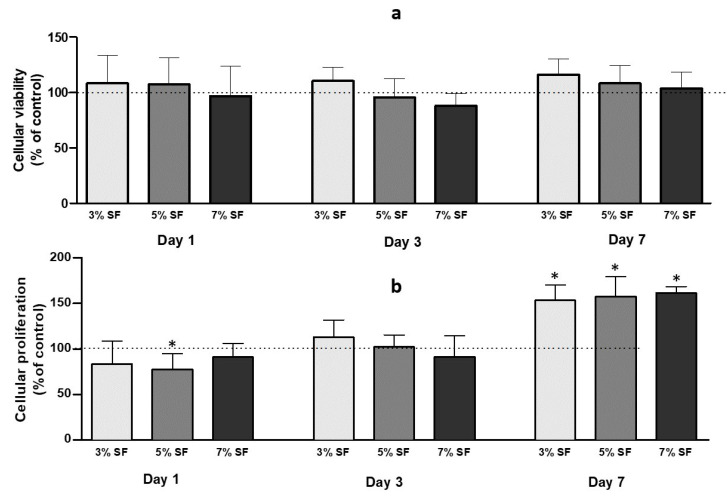
(**a**) Cell viability after MTT assay, and (**b**) quantification of cell proliferation after BrdU assays of a control group of HDF cells and cells cultured with aerogel particles (*p* < 0.05). * The differences from the negative control (cell growth) of each time-point were significant at *p* < 0.05.

The cell viability of the aerogels was evaluated using the MTT assay. After 24 h of incubation, the results showed that all aerogels maintained cell viability up to 70%. No significant differences were observed between the cells in contact with aerogel particles and the control group (Figure 5). 

To evaluate the effect of SF-Ap on cellular proliferation, HDF cells were subjected to a BrdU incorporation assay. On day 1, a slight decrease in cell proliferation was observed in the 5% SF group compared to the control group. Nevertheless, this decrease was not statistically significant. By day 7, a remarkable increase in cellular proliferation was observed across all SF-Ap formulations. Significant differences were found when comparing the SF aerogel groups to the control group, indicating the effect of SF-Ap on stimulating cellular proliferation over time. These findings highlight the potential of SF-Ap to enhance cellular proliferation and suggest their suitability for promoting tissue regeneration and growth.

**Figure 6:**
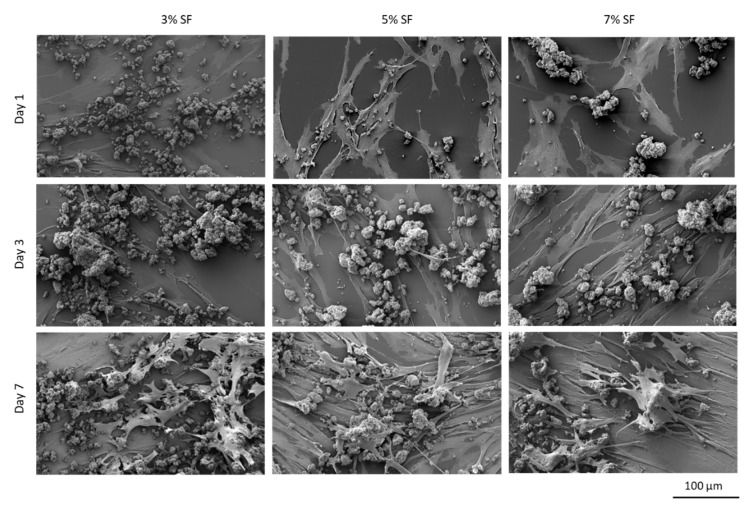
SEM micrographs of HDF’s cell cultures in contact with SF-Ap for 1, 3, and 7 days. Magnification 1500×.

**Figure 7:**
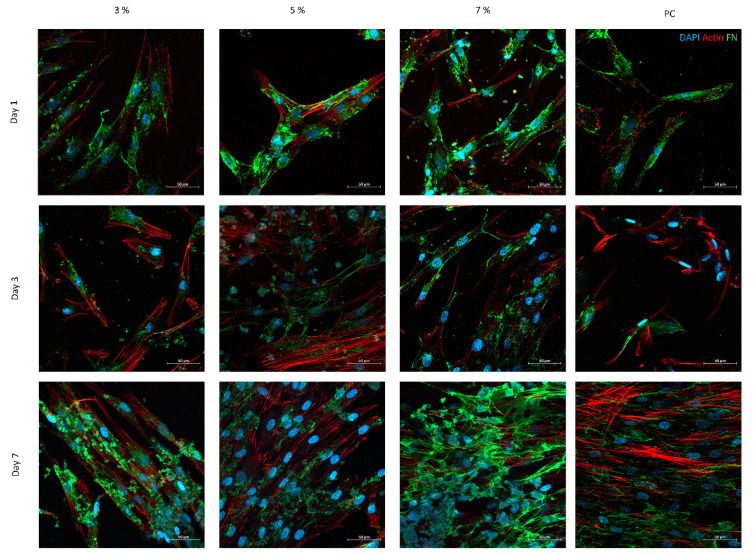
Confocal microscopy images were used to visualize the morphology, distribution, and growth of the nuclei, cytoskeleton, and fibronectin of human dermal fibroblasts (HDFs) when cultured in direct contact with the produced sample SF-Ap for 1, 3, and 7 days of culture (scale bars: 50 µm).

The different SF-Ap were analyzed by SEM and confocal microscopy after cell seeding and cultured up to 7 days (Figure 6 and Figure 7, respectively).

SEM images demonstrate the positive interaction of the cells with the particles during cell culture, showing that—for all the conditions—cells present the typical fibroblastic morphology (spindle and elongated shape). These images support the results obtained for the proliferation tests, showing that the cellular interaction with 7% SF particles is enhanced. The interconnectivity of the pores on the SF-Ap aerogel particles can also be regarded as a contributing factor to the enhanced cell proliferation and cell adhesion over time, since nutrients and oxygen can freely circulate through the particles, ensuring cell nutrition.

Confocal micrographs, performed for the same time periods of cell culture, show a clear increase of the cell number with culture time, correlating well with SEM observations. The presence of FN deposition was investigated since it emerged among the ECM protagonists as the most pertinent representative key actor [53]. In fact, FN is involved in numerous physiological processes—namely, cell adhesion to the ECM—and contributes to phagocytosis regulation, wound healing, cell proliferation, and differentiation [53]. Regarding the images on the first day, it is possible to observe for all conditions the strong interaction between cells and the produced particles. Moreover, all conditions present FN deposition in a fibrillary form. After 7 days of culture, the presence of cytoskeleton, which provides a structural framework, facilitates intracellular transport, supports cell junctions, and transmits signals about cell contact, adhesion, and motility, is lower in the samples with 3% and 5% of SF, probably due to the low concentration of SF. On the other hand, structures with 7% SF present a complex cytoskeleton with several cell junctions forming a network. Some studies have demonstrated that the porous nature of these structures exerts a significant influence on cell behavior [54,55]. Interestingly, the porosity and mesoporous structure of these particles closely resemble those of other formulations. This observation suggests that SF may provide high adhesion sites and therefore a better surface functionality for facilitating cell adhesion. This hypothesis indicates a direct correlation between SF concentration and cell adhesion. Nevertheless, in all conditions, the FN has been deposited by the cells in a fibrillar form and, compared with the actin cytoskeleton location, FN distribution followed cellular organization. 

The possibility for integration of relevant therapeutic molecules within its matrix due to the mild processing technology can further amplify the potential of the herein developed SF aerogel system and its bioactivity, thus substantiating the potential of this porous material as a drug delivery system [19].

## 4. Conclusions

SF aerogel particles with enhanced biological properties were obtained by the addition of ethanol during gel formation as they induced a stable β-sheet conformation followed by scCO_2_-assisted drying. Higher SF concentrations resulted an increased viscosity of the SF precursor solution which allowed for the formation of larger particles with broader size distribution. The aerogel particles presented a mesoporous interconnected structure, facilitating cell migration and nutrient diffusion. The high porosity and roughness of the particle surface play a crucial role in promoting a robust interaction with cells. SF aerogel particles displayed significant antioxidant activity and sustained degradation in the presence of protease, making them promising systems for wound healing and tissue regeneration applications. SF aerogels were highly biocompatible, promoting cell viability, attachment, and proliferation, indicating their potential for various biomedical applications—particularly in tissue healing and regeneration. Future research will focus on exploring the efficacy of these materials as drug carriers for the treatment of chronic wounds, including swelling studies and in vitro tests with different type of cells that play a role in wound healing and pre-clinical assays. 

## Figures and Tables

**Table 1:** Particle size distribution parameters, namely Dv10, Dv50, Dv90, and span, were assessed for alcogel and aerogel SF particles using laser diffraction analysis, while microscopy analysis was employed exclusively for aerogel SF particles.

	SF Alcogel Particles				SF Aerogel Particles								
	Mastersizer Analysis				Mastersizer Analysis				Microscopy Analysis				
	Dv10	Dv50	Dv90	Span ^1^	Dv10	Dv50	Dv90	Span ^1^	Average Diameter (µm)	Dv10	Dv50	Dv90	Span ^1^
	(µm)	(µm)	(µm)		(µm)	(µm)	(µm)			(µm)	(µm)	(µm)	
3% SF	11.8 ± 0.1	23.7 ± 0.2	43.1 ± 0.5	1.3	11.3 ± 0.4	32.4 ± 2.6	235.0 ± 74.8	6.9	22.3 ± 8.48	12.1	21.7	29.1	0.78
5% SF	14.2 ± 0.0	31.0 ± 0.1	59.1 ± 0.7	1.4	10.9 ± 0.1	28.8 ± 0.7	66.6 ± 4.3	1.9	27.1 ± 13.98	13.9	23.3	45.0	1.34
7% SF	12.6 ± 0.1	31.4 ± 0.1	81.8 ± 1.5	2.2	9.75 ± 0.0	35.6 ± 0.9	304.0 ± 23.9	8.3	29.4 ± 12.0	15.9	26.8	47.4	1.18

^1^ value obtained by applying Equation (1).

**Table 2:** Characterization of SF-Ap. Notation: $\rho_{skel}$, skeletal density using helium pycnometry; $\rho_{env}$, envelope density calculated according to the methodology; ε, porosity calculated using Equation (2).

Particles	$\rho_{skel}$ (g/cm^3^)	$\rho_{env}$	ε ^1^ (%)
3% SF	1.25 ± 0.03	0.07 ± 0.002	94 ± 0.19
5% SF	1.33 ± 0.02	0.08 ± 0.016	94 ± 1.21
7% SF	1.22 ± 0.02	0.08 ± 0.013	93 ± 1.03

^1^ value obtained by applying Equation (2).

**Table 3:** Textural properties evaluated by nitrogen adsorption–desorption test of the SF-Ap prepared with concentration (3%, 5%, and 7% SF). Notation: aBET, specific surface area by the BET method; VP,BJH, overall specific pore volume obtained by the BJH-method; DP,BJH, mean pore diameter by the BJH-method; V_mes_, specific mesopore volume; V_mac_, specific macropore volume.

Particles	aBET (m^2^/g)	VP,BJH (cm^3^/g)	DP,BJH (nm)	V_mes_ (cm^3^/g)	V_mac_ (cm^3^/g)
3% SF	237 ± 12	1.90 ± 0.44	27.25 ± 2.39	1.43 ± 0.33	13.05 ± 0.33
5% SF	326 ± 16	2.33 ± 0.43	24.46 ± 1.40	1.75 ± 0.29	11.41 ± 0.29
7% SF	204 ± 10	1.53 ± 0.35	24.76 ± 2.52	1.16 ± 0.26	11.32 ± 0.26

## Data Availability

Data sharing is not applicable.
